# Attention-Related Brain Activation Is Altered in Older Adults With White Matter Hyperintensities Using Multi-Echo fMRI

**DOI:** 10.3389/fnins.2018.00748

**Published:** 2018-10-18

**Authors:** Sarah Atwi, Arron W. S. Metcalfe, Andrew D. Robertson, Jeremy Rezmovitz, Nicole D. Anderson, Bradley J. MacIntosh

**Affiliations:** ^1^Heart and Stroke Foundation Canadian Partnership for Stroke Recovery, Sunnybrook Research Institute, University of Toronto, Toronto, ON, Canada; ^2^Department of Medical Biophysics, University of Toronto, Toronto, ON, Canada; ^3^Centre for Youth Bipolar Disorder, Sunnybrook Research Institute, University of Toronto, Toronto, ON, Canada; ^4^Department of Family and Community Medicine, Sunnybrook Health Sciences Centre, Toronto, ON, Canada; ^5^Department of Psychiatry and Psychology, University of Toronto, Toronto, ON, Canada; ^6^Rotman Research Institute, Baycrest Centre, University of Toronto, Toronto, ON, Canada

**Keywords:** white matter hyperintensities, small vessel disease, attention, multi-echo, fMRI, BOLD

## Abstract

Cognitive decline is often undetectable in the early stages of accelerated vascular aging. Attentional processes are particularly affected in older adults with white matter hyperintensities (WMH), although specific neurovascular mechanisms have not been elucidated. We aimed to identify differences in attention-related neurofunctional activation and behavior between adults with and without WMH. Older adults with moderate to severe WMH (*n* = 18, mean age = 70 years), age-matched adults (*n* = 28, mean age = 72), and healthy younger adults (*n* = 19, mean age = 25) performed a modified flanker task during multi-echo blood oxygenation level dependent functional magnetic resonance imaging. Task-related activation was assessed using a weighted-echo approach. Healthy older adults had more widespread response and higher amplitude of activation compared to WMH adults in fronto-temporal and parietal cortices. Activation associated with processing speed was absent in the WMH group, suggesting attention-related activation deficits that may be a consequence of cerebral small vessel disease. WMH adults had greater executive contrast activation in the precuneous and posterior cingulate gyrus compared to HYA, despite no performance benefits, reinforcing the network dysfunction theory in WMH.

## Introduction

Growing neuroanatomical and behavioral evidence suggests CSVD contributes to subclinical neurological deficits, but the underlying neurofunctional correlates remain unclear. CSVD is a prevalent disorder with detrimental effects on the cerebrovasculature, brain tissue, cognition, and behavior (Wardlaw et al., 2013). This insidious disease increases the risk of stroke and dementia (Snowdon et al., 1997; Vermeer et al., 2003; Barnes et al., 2013) and is responsible for myriad alterations in arteriolar, capillary and venular beds (Pantoni, 2010). A consensus of neuroanatomical imaging identifies WMH of presumed vascular origin, seen on T2-weighted MRI, as the most common hallmark of CSVD (Rost et al., 2010; Wardlaw et al., 2013). WMH impact not only the physiology of white matter (Makedonov et al., 2013) but also the anatomy and physiology of gray matter (O’Sullivan et al., 2002; Zheng et al., 2006; Bastos-Leite et al., 2008; Fu et al., 2014; Wagner et al., 2015; Shi et al., 2016).

Cognitive assessments reveal detrimental associations between CSVD and multiple behavioral domains. WMH volume is inversely related to cognitive function, globally (de Groot et al., 2000; Schmidt et al., 2004; Kerchner et al., 2012), and to processes of attention in particular (Ishikawa et al., 2012), including executive function (Breteler et al., 1994; Wright et al., 2008; Zheng et al., 2012). Attention-related dysfunction can be identified using the attention network test, which elicits brain-wide cognitive engagement using a flanker task with graded difficulty (Fan et al., 2005, 2002). Functional neuroimaging techniques that probe brain and behavior relationship in tasks of increasing difficulty may play an important role in advancing our understanding of subclinical features of WMH.

CSVD has been linked to lower functional coupling and coherence, using electroencephalography, which was reflected in lower speed of information processing (Dey et al., 2016). BOLD is a functional MRI (fMRI) approach which can probe CSVD by characterizing neurovascular signal patterns that may relate to impaired dorsal attention, default mode and fronto-parietal control networks (Spreng et al., 2013). To date, however, task-based fMRI among adults with WMH has produced conflicting findings; both increased and decreased activation are reported in attentional neural networks (Nordahl et al., 2006; Venkatraman et al., 2010; Hedden et al., 2012; Lockhart et al., 2015; Gold et al., 2017). Despite the utility of BOLD fMRI, challenges exist in the context of aging that influence signal-to-noise ratio and specificity of activation, be they physiological, neurovascular, or metabolic (Huettel et al., 2001). BOLD fMRI analysis is mature, with strategies in place to isolate and minimize physiological sources of non-interest to increase detection of neurovascular activation (Hu et al., 1995; Biswal et al., 1996; Wowk et al., 1997; Glover et al., 2000; Birn et al., 2006; Caballero-Gaudes and Reynolds, 2016). An important gap in the literature involves the limited evaluation of targeted data denoising techniques in fMRI studies probing cerebrovascular pathology, which is likely due to a greater focus on the structural changes associated with cerebrovascular dysfunction, rather than functional changes.

Improved paradigm, acquisition, and data-scrubbing strategies are likely to advance fMRI in CSVD research. The current study incorporates each of these elements as part of the experimental design, namely multi-echo fMRI during a modified attention network test (Fan et al., 2005). Multi-echo fMRI aggregates multiple image volumes to improve image quality (Posse et al., 1999; Poser et al., 2006; Kundu et al., 2013), reduces susceptibility-related signal loss (Kirilina et al., 2016), enhances anatomical-functional registration (Kundu et al., 2015), and filters high frequency artifacts (Olafsson et al., 2015). By acquiring echo-planar images at multiple echo times, it is possible to reconstruct images in combination to accentuate particular image contrasts, although a weighted-echoes approach is most common (Posse et al., 1999). Multi-echo fMRI outperforms conventional fMRI during rapid event-related tasks (Gonzalez-Castillo et al., 2016; Lombardo et al., 2016). This is likely due to improvements in statistical power of task-related data, both at the individual and group level analysis, and by mitigating motion artifact signals, a central hindrance to producing robust BOLD data (Buur et al., 2008, 2009; Witt et al., 2016; Kundu et al., 2017).

The primary purpose of the current study was to compare widespread task network and executive network activation in response to an attention-demanding task across three groups of adults. We hypothesized that multi-echo fMRI would show marked activation and behavioral performance differences in older adults with WMH relative to age-matched healthy adults and younger healthy adults. Given that multi-echo fMRI has not been used in CSVD to date, we also consider its performance relative to the established Retrospective Correction of Physiological Motion Effects (RETROICOR) method (Glover et al., 2000).

## Materials and Methods

### Participants

This study recruited community-dwelling individuals, comprising of healthy younger individuals (HYA), HOAs with minimal WMH lesion burden (HOA), and older adults with moderate to severe lesion burden (WMH). Most participants were recruited through community advertising. A portion of WMH adults was recruited through a Family Medicine clinic mail-out, which targeted patients with one or more vascular risk factors. Exclusion criteria included contraindications to MRI, a score below 21 on the Montreal Cognitive Assessment (MoCA), and a self-reported history of type 2 diabetes requiring insulin, cardiopulmonary illness, stroke, or dementia. WMH severity was indexed by the Fazekas rating scale from 0 to 3 using the following rubric: 0 – no periventricular or deep white matter WMH; 1 – pencil-thin lining or capped periventricular WMH and/or punctate foci in deep white matter; 2 – smooth periventricular WMH halo and/or beginning of deep WMH confluence; and 3 – irregular periventricular signal extending to deep white matter and/or large deep white matter confluence (Fazekas et al., 2002). Those with Fazekas ≥ 2 were assigned to the WMH group. All older participants performed the Digit Symbol Coding Test (DSCT), as well as the MoCA. This study was approved and carried out in accordance with the recommendations of the Sunnybrook Healthy Sciences Centre Research Ethics Board with written informed consent from all subjects. All subjects gave written informed consent in accordance with the Declaration of Helsinki. The Sunnybrook Healthy Sciences Centre Research Ethics Board approved the protocol. A total of 65 participants, including 19 HYA [10 female, age (mean ± SD) 25 ± 1 years], 28 HOA (17 female, 70 ± 6 years), and 18 WMH (10 female, 72 ± 5 years) were MRI eligible and agreed to participate, received brain MRI, and underwent cognitive testing. The groups were well matched in terms of years of education (*F* = 0.52, df = 2,64, *P* = 0.47), sex (χ^2^ = 0.8, df = 2,64, *P* = 0.7). The older groups were age matched (*W* = 193, *P* = 0.19) and MoCA total score was not different (*W* = 301, *P* = 0.083). Additional demographic details, including a group difference in WMH volumes (*W* = 24, *P* < 0.001) are provided in **Table 1**.

**Table 1:** Group demographics and clinical characteristics.

	HYA, *n* = 19	HOA, *n* = 28	WMH, *n* = 18
Age, years	25 ± 3	70 ± 6	72 ± 5
Sex, F:M	10:9	17:11	10:8
Education, years	17 ± 2	17 ± 3^a^	17 ± 2
MoCA, total score (range)	–	26 ± 2 (23–30)^a^	25 ± 2 (22–29)^b^
DSCT total score (range)	–	67 ± 14 (14–89)	56 ± 11(27–70)^b^
WMH volume, ml	–	0.71 ± 0.6^b^	9.21 ± 0.6
Hypertension, count (%)	0	0(0)	4(22)
Type II Diabetes Mellitus, count (%)	0	1(4)	1(6)


*Data are expressed as mean ± standard deviation or n (%). MoCA, Montreal Cognitive Assessment; DSCT, Digit Symbol Coding Test; WMH, white matter hyperintensities; a, *n* = 27; b, *n* = 17.*

### MRI Acquisition

Images were acquired using a 3T scanner (Achieva, Philips Healthcare, Best, Netherlands) with an 8-channel head coil receiver. High-resolution 3D T1-weighted anatomical images were acquired using the following parameters: repetition time (TR) = 9.5 ms, echo time (TE) = 2.3 ms, 140 slices, flip angle = 8°, voxel dimensions = 0.63 × 0.63 × 1.2 mm. Fluid-attenuated inversion recovery (FLAIR) images were acquired in 2D with T2-weighting to assess WMH lesions using the following parameters: TR = 9000 ms, TE = 125 ms, 52 slices, flip angle = 90°, voxel dimensions = 0.43 × 0.43 × 3 mm. The multi-echo BOLD acquisition was 7 min and 5.5 s in duration (TR = 2300 ms, 185 volumes) and involved the following parameters: three consecutive echoes per volume at TE = 13.82, 35.35, and 56.89 ms, 28 slices, flip angle = 70°, and voxel dimensions = 2.88 × 2.88 × 4 mm. Heart rate and breathing rate were monitored using pulse oximetry and respiratory bellows, respectively.

### WMH Volume Quantification

Gray matter, white matter and cerebrospinal fluid were segmented on the T1-weighted image using FMRIB Automated Segmentation Tool (Zhang et al., 2001). WMH were segmented on the FLAIR images using in-house software, a fuzzy lesion extractor (Gibson et al., 2010). Two experienced and blinded raters [Sarah Atwi and Andrew D. Robertson] performed Fazekas ratings. Inter-rater reliability was 87%, and discrepancies in scoring were discussed to achieve consensus. A FLAIR image was not acquired for one older adult; however, they were classified as an HOA due to lack of WMH features in the T1-weighted image.

### Task fMRI

The attention network test is a flanker paradigm that probes attention and psychomotor speed (Fan et al., 2005). The event-related task design was implemented using E-Prime v1.2 (Psychology Software Tools, Pittsburgh, PA, United States) and consisted of 12 in-scanner practice trials with feedback (requiring 100% accuracy on the final 6 trials) and 120 experimental trials during fMRI. In addition, participants performed a shortened practice test using a keyboard response triggers immediately prior to MRI scanning. Participants used right and left hand, index finger response triggers to identify the direction of the middle arrow (i.e., respond using the same hand as the direction of the middle arrow), while four flanking arrows pointed congruently, or incongruently in the direction of the target (e.g., congruent condition: →→→→→, ←←←←←; incongruent condition: →→←→→, ←←→←←). The arrows were presented above or below a centralized fixation cross, which appeared for 150 ms at the onset of each trial, and were counterbalanced across conditions. On two-thirds of trials, a warning cue appeared for 400 ms, above, below, or superimposed upon the fixation cross; all other trials received continued 400 ms of fixation. At 550 ms post-onset, the flankers and target were presented for 1550 ms. The post-target fixation period was 660 ms. Inter-trial intervals ranged from 0 to 7360 ms (mean = 900 ms) during which the fixation was always present. The event-related contrast used low-level fixation as the baseline to test widespread task network engagement. A higher-level *executive* contrast was studied by analyzing incongruent task activation versus low-level fixation baseline minus congruent task activation versus low-level fixation baseline. With- and without-cue flanker conditions were included in the *executive* contrast to maximize the number of trials.

### Functional Image Post-processing and Time Series Analysis

Multi-echo BOLD data were combined using a weighting scheme first described by Posse et al. (1999), and later detailed by Poser et al. (2006). This approach weights the signal intensities (S) from each echo time image by using the summation of data acquired at different echo times (TE_n_) at each TR multiplied by a weighted function that uses the derived relaxation time ($T_{2}^{*}$):

$$\mathrm{Weighted}\;\mathrm{Echoes}\;\mathrm{Signal}=\sum_{n=1}^{N}S(TR,\;TE_{n})\left(\frac{TE_{n}}{T_{2}^{*}}\exp\left(-\frac{TE_{n}}{T_{2}^{*}}\right)\right)$$

For comparison, we also processed the second echo (TE = 35.35 ms) alone to emulate a single-echo analysis, which included a retrospective correction of physiological motion effects (RETROICOR) (Glover et al., 2000). FMRI processing was carried out on the weighted-echo, RETROICOR, and uncorrected single-echo (TE = 35.35) versions of the fMRI data, as described in Section “BOLD Processing Comparison.”

We used FMRIB software library Version 5.0 for fMRI analysis. The Linear Image Registration Tool (FLIRT) was used to correct for head motion, the Brain Extraction Tool was used to extract brain from skull (Smith, 2002), and the Expert Analysis Tool (FEAT) was used to spatially smooth with a 6-mm Gaussian kernel, intensity normalize by a single multiplicative factor, and temporally filter (high-pass) equivalent to 50 s (Jenkinson et al., 2002). Functional data were registered to the T1-weighted image using 6 degrees of freedom (Greve and Fischl, 2009). Registration of the T1-weighted image to Montreal Neurological Institute space with 2-mm isotropic resolution was carried out using FLIRT (Jenkinson and Smith, 2001; Jenkinson et al., 2002) and was refined using FNIRT non-linear registration (Andersson et al., 2007). Participants’ head motion was inspected by the mean frame-wise displacement and those with a mean displacement below 0.4 mm were included in the analysis.

Time-series statistical analysis was carried out using FEAT to create a general linear model with local autocorrelation correction. The behavioral task patterns were convolved with a double gamma hemodynamic response function. The main contrasts of interest include only correct trials during: (1) *all versus baseline* conditions and (2) *executive* contrast (i.e., incongruent versus congruent conditions).

### BOLD Processing Comparison

**FIGURE 1:**
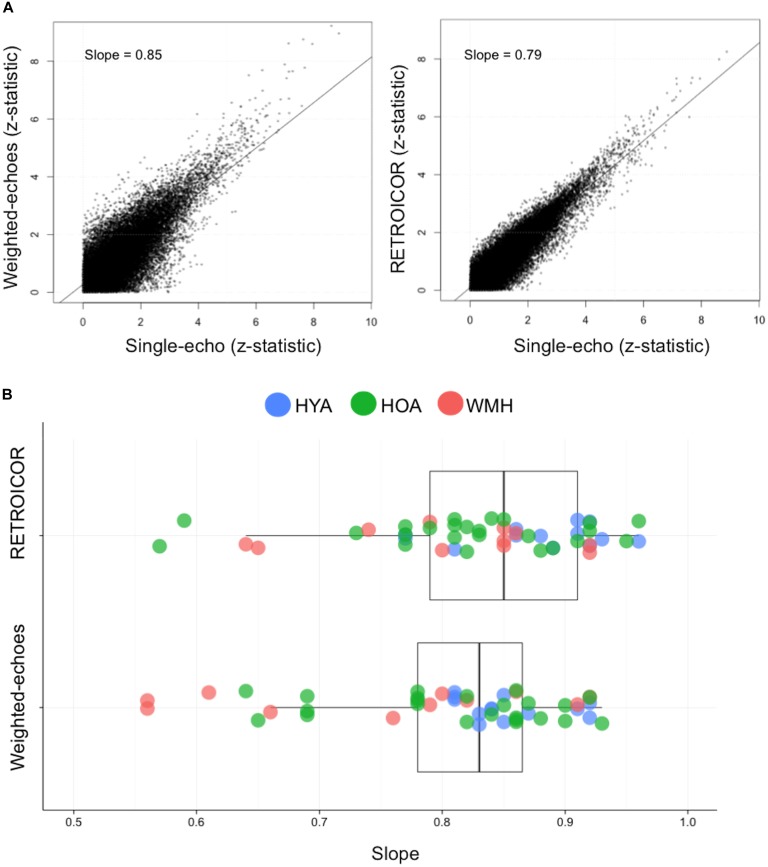
Relationship between single-echo functional image acquisition with RETROICOR and weighted-echoes post-processing methods. **(A)** The comparison between single-echo acquisition and functional image with RETROICOR data or weighted-echoes technique using z-stats from each voxel from a representative individual. The coefficient of determination was lower for weighted-echoes (*P* < 0.001), which demonstrates RETROICOR had more shared variance with single-echo processing method. **(B)** The slope of RETROICOR versus single-echo and the slope of weighted-echoes versus single-echo were both less than one (*P* < 0.001), indicating single-echo acquisition z-stats were relatively greater than after RETROICOR or weighted-echoes processing was applied. The two sets of slope estimates were not significantly different (*P* = 0.1).

To examine the utility of multi-echo fMRI, we compared the weighted-echoes and single-echo RETROICOR approaches to the uncorrected single-echo data. The within-subject comparisons were based on Z-statistic maps from the *all versus baseline* task contrast, without adjustment for reaction time. We calculated the slope of a line of best fit through voxel-wise data from each post-processing approach versus the uncorrected single-echo reference for each participant (i.e., weighted-echoes Z-stat map versus single-echo Z-stat map; RETROICOR Z-stat map versus single-echo Z-stat map; representative participant in **Figure 1A**). A slope of 1 signifies the post-processing approach yields equivalent *Z*-values compared to the uncorrected single-echo analysis. A slope less than 1 indicates *Z*-values are lower than an uncorrected single-echo analysis, and a slope above 1 would signify higher Z-values in this voxel-by-voxel comparison. *R*^2^ values from the linear association between RETROICOR and single-echo and between weighted-echoes and single-echo were compared. Group differences in slope and *R*^2^ values for both method comparisons were assessed. Given that multi-echo fMRI and RETROICOR are able to reduce spurious signals, this analysis would help support hypothesis testing using the weighted-echoes data.

### Statistical Analysis

Group differences for sex, hypertension, and diabetes mellitus were assessed using Pearson’s Chi-squared test. Group differences for education, task reaction time, and task accuracy were assessed using one-way ANOVA and *post hoc* pairwise comparison with Tukey’s adjustment for multiple comparisons. Differences between HOA and WMH for age, DSCT, MoCA, and WMH volume were assessed using a Wilcoxon rank sum test. These tests were performed using R software (V3.1.1) and significant differences were inferred at *P* < 0.05.

FMRI processing methods were compared using a Welch two sample *t*-test of the slope and *R*^2^ values from the linear associations between RETROICOR and single-echo Z-stats, and between from the weighted-echoes and single-echo Z-stats. Differences between each method and the single-echo method were assessed using a one-sample *t*-test comparing the slopes to 1. Group differences in slope and *R*^2^ values were analyzed using a one-way ANOVA and *post hoc* pairwise comparison with Tukey’s adjustment for multiple comparisons.

Using the weighted-echoes fMRI, a group analysis was performed using only correct trials during the task. We identified regions of interest based on a voxel-level threshold of Z > 2.32 (*P* < 0.01) followed by a cluster-level threshold of *P* < 0.05. This approach accounts for family wise error and was implemented as a mixed effects model using FMRIB’s Local Analysis of Mixed Effects (stage 1 + 2), which estimates the higher-level parameter estimates and mixed effects variance for group comparisons (Beckmann et al., 2003; Woolrich et al., 2004). We tested for an effect of group and subsequent paired group differences after inspecting single group average maps using FEAT. In addition to uncorrected results, we adjusted brain activation for performance using mean of correct trial reaction times during *all conditions*. Comparison of reaction times and percent BOLD signal changes was studied during the *all versus baseline, incongruent*, and *congruent* conditions. Signal within significant clusters from the unadjusted all-group’s average maps of each condition was independently compared to respective reaction times and accuracy using Pearson-product moment correlation.

## Results

### Group Characteristics

Clinical and demographic information for participants are presented in **Table 1**. The WMH group had a lower DSCT scores (*W* = 376.5, *P* = 0.0.001) and higher prevalence of hypertension (*P* = 0.04) compared to HOA.

### Multi-Echo Versus RETROICOR Comparison

Seven WMH, 4 HOA, and 6 HYA were omitted from the methods comparison due to incomplete heart rate and ventilation rate log files, precluding the RETROICOR processing. Relative to the uncorrected single-echo fMRI, voxel-wise Z-stats from the weighted-echo method produced a slope of 0.80 ± 0.1, and the RETROICOR method produced a slope of 0.83 ± 0.09; these slopes were not different from one another (*t* = -1.6, df = 91.7, *P* = 0.1) (**Figure 1**). The slopes were, however, less than unity, indicating both approaches tend to reduce Z-stat values (weighted-echo: *t* = -13.0, df = 47, *P* < 0.001; RETROICOR: *t* = -12.9, df = 47, *P* < 0.001). *R*^2^ values from the association between the weighted-echo and single-echo Z-stats were lower than those from the association between RETROICOR and single-echo Z-stats (*t* = -4.0, *P* < 0.001). Weighted-echoes versus single echo slope were different between groups (*F* = 3.1, df = 2,47, *P* = 0.05), where HYA had higher slopes than WMH (*P* = 0.04), but *R*^2^ values did not differ between groups (*F* = 2.8, df = 2,47, *P* = 0.07). RETROICOR versus single-echo slopes did not differ between groups (*F* = 2.3, df = 2,47, *P* = 0.1), but *R*^2^ values were significantly different (*F* = 4.1, df = 2,47, *P* = 0.02), in that HYA had higher *R*^2^ compared to WMH (*P* = 0.02). The weighted-echo maps showed lower spatial dispersion of signal comparative to RETROICOR (see **Supplementary Figure S1**).

### Behavioral Results

**FIGURE 2:**
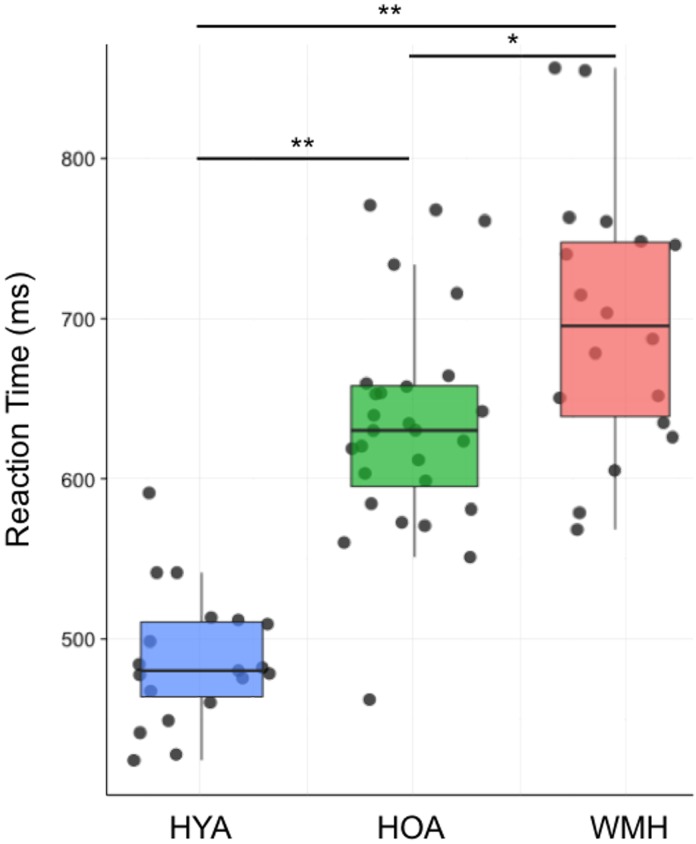
Average reaction time group differences across all trials of the fMRI attention network task. Average reaction time during both *congruent* and *incongruent* conditions indicate WMH had reduced performance compared to both HOA and HYA, and HYA performed better than HOA. ^∗^ < 0.01 and ^∗∗^ < 0.001.

Across all trials, accuracy was not different between groups (**Table 2**; *F* = 0.4, df = 2,64, *P* = 0.7). When considering only the correct trials, there was a main effect of group on reaction time (*F* = 49.2, df = 2,64, *P* < 0.0001) (**Figure 2**). HYA had shorter reaction times than both HOA and WMH (*P* < 0.001), and HOA had shorter reaction times than WMH (*P* < 0.01) (**Table 2**). Similarly, group differences in reaction time were observed for all correct *congruent* and *incongruent* tasks (i.e., including trials with- and without- cues) (congruent: *F* = 45.04, df = 2,64, *P* < 0.001; incongruent: *F* = 49.2, df = 2,64, *P* < 0.001) where HYA had lower reaction times compared to HOA and WMH (congruent: *P* < 0.001; incongruent: *P* < 0.001), and HOA had lower reaction times compared to WMH (congruent: *P* = 0.02; incongruent: *P* = 0.005). Accuracy did not differ between groups (congruent: *F* = 2.3, df = 2,64, *P* = 0.1; incongruent: *F* = 0.4, df = 2,64, *P* = 0.7). *Executive* contrast of *incongruent* minus *congruent* reaction times did not differ between groups (*F* = 2.3, df = 2,64, *P* = 0.1).

**Table 2:** Reaction time and accuracy of attention network test during fMRI scan.

	HYA	HOA	WMH
**All conditions**			
Reaction time, ms	487.2 ± 41.5	634.8 ± 69.5	698.3 ± 83.5
Accuracy, %	98.6 ± 1.1	98.8 ± 1.9	98.3 ± 1.8
**Congruent**			
Reaction time, ms	456.6 ± 39.0	600.7 ± 71.0	657.8 ± 83.2
Accuracy, %	99.4 ± 1.0	99.3 ± 1.3	98.4 ± 2.2
**Incongruent**			
Reaction time, ms	517.9 ± 46.2	668.9 ± 71.3	738.8 ± 86.7
Accuracy, %	97.7 ± 1.9	98.3 ± 2.6	98.2 ± 1.8
**Executive contrast**			
Reaction time, ms	183.9 ± 60.6	204.6 ± 91.1	243.1 ± 95.1
Accuracy, %	–	–	–


*Accuracy % is the accuracy percentage per trial.*

### All Versus Baseline Condition Weighted-Echoes fMRI Group Activation Differences

**FIGURE 3:**
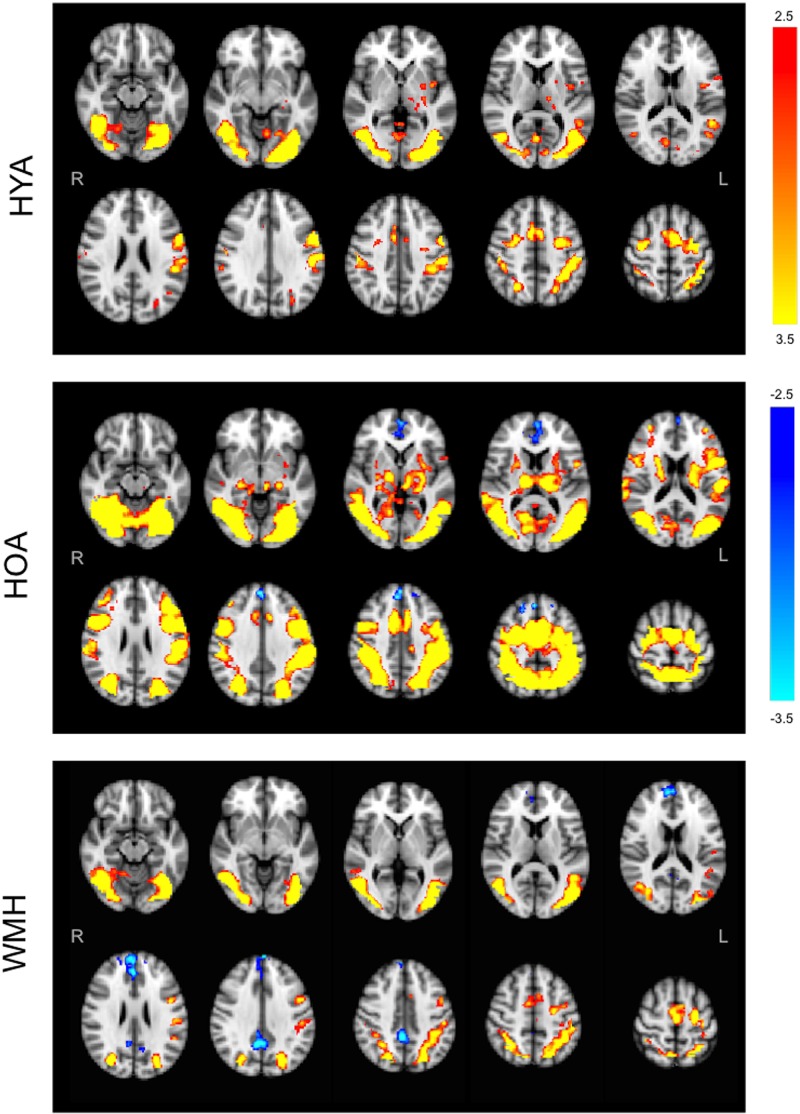
Group average statistical maps of BOLD fMRI signal for *all versus baseline* condition. HYA activated bilateral sections of the visual and attention networks like the anterior cingulate gyrus, occipital cortex, left insula, parietal opercular, and parietal lobes, and supplementary motor cortex, with no evidence of deactivation. HOA activated the visual and attention networks including fronto-parietal areas, insula, and lateral occipital cortices, with deactivation in the frontal pole, cingulate and paracingulate. WMH demonstrated tightly localized activity in the attention system (middle frontal), with deactivation throughout interoceptive network nodes (frontal regions and precuneus). Primary threshold for all maps was *Z* = 2.32 and cluster-correction threshold was *P* = 0.05. Warm colors, activation relative to fixation. Cool colors, deactivation relative to fixation.

**FIGURE 4:**
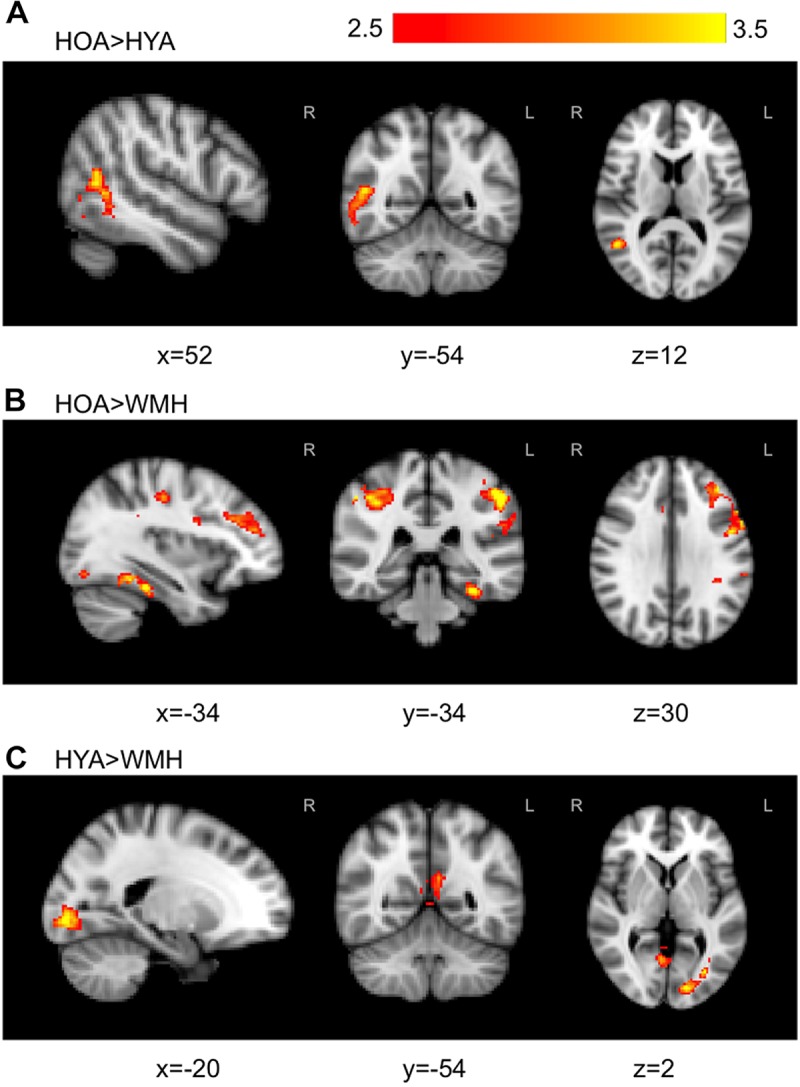
Group comparisons with statistically significant regions of signal differences found in WMH versus HOA and HYA during *all versus baseline* condition. **(A)** The contrast of HOA and HYA revealed HOA exhibited higher activation in late stage visual attention processing circuits in temporal and occipital fusiform. **(B)** In contrast, HOA demonstrated higher activation than WMH in cognitive attention loci including the fronto-parietal cortical areas. **(C)** WMH had reduced activation compared to HYA in the precuneous, cingulate, and occipital areas. Primary threshold for all maps was *Z* = 2.32 and cluster-correction threshold was *P* = 0.05. Warm colors, z-stat intensity differences.

The *all versus baseline* condition activation maps, adjusted for correct-trial reaction time, revealed a network of activation in frontal, subcortical, parietal, and occipital regions (**Figure 3** shows the mean activation per group). The HOA group had a greater spatial extent of activation compared to the other groups (**Figure 3**). Further, group differences in peak activation were observed (omnibus ANOVA: *P* < 0.05). In pairwise comparisons, the HOA group had greater activation than HYA in the occipital lobe, inferior temporal gyrus, middle temporal gyrus, and angular gyrus (**Table 3** and **Figure 4A**). The HOA group also had greater activation compared to WMH in a fronto-temporal region comprising the planum temporale, temporal gyrus, frontal pole, parietal lobe, supramarginal, postcentral, precentral, and middle and frontal gyri, as well as a parieto-occipital region comprising the parietal operculum cortex, lateral occipital cortex, and precuneous cortex (**Table 3** and **Figure 4B**). Moreover, pair-wise comparisons showed that the HYA group had greater activation compared to WMH in an occipital region comprising the calcarine cortex, lingual gyrus, and occipital cortex, pole, and fusiform gyrus, as well as a parieto-occipital region comprising the precuneous, and posterior cingulate gyrus, and right cuneal cortex (**Table 3** and **Figure 4C**). No clusters were found for the reverse contrasts, i.e., the WMH group did not have greater activation than either HOA or HYA. Reaction-time-unadjusted *all versus baseline* condition average activation maps for WMH and HOA were comparable to the reaction-time-adjusted results. An HOA versus HYA contrast was absent in the unadjusted analysis.

**Table 3:** Peak brain activation during all conditions versus baseline and executive contrasts.

Area		Hemisphere	MNI coordinates			Cluster size (voxels)	Z_peak_
					
			*x*	*y*	*z*		
**All versus baseline condition**							
Main effect of HOA (HOA > HYA)							
	Temporal occipital fusiform cortex, inferior temporal gyrus, inferior lateral occipital cortex, middle temporal gyrus, angular gyrus.	R	40	-48	-22	569	3.62
Main effect of HOA (HOA > WMH)							
	Superior parietal lobule, anterior supramarginal gyrus, postcentral gyrus, precentral gyrus, middle frontal gyrus, inferior frontal gyrus, planum temporale, posterior superior temporal gyrus, frontal pole, parietal operculum cortex.	L	-60	8	28	2064	4.82
	R precentral gyrus, R postcentral gyrus, R supramarginal gyrus, L and R superior parietal lobe, R superior lateral occipital cortex, L and R precuneous cortex, R parietal operculum cortex.	L, R	38	-46	54	1609	3.94
	Inferior lateral occipital cortex, occipital fusiform gyrus, temporal occipital fusiform cortex, lingual gyrus, inferior temporal gyrus, middle temporal gyrus.	R	40	-50	-22	874	4.09
	Occipital pole, inferior lateral occipital cortex, lingual gyrus, posterior temporal fusiform cortex.	L	-6	-96	-4	621	4.93
	L and R Paracingulate gyrus, R anterior cingulate gyrus, R middle frontal gyrus, L and R juxtapositional lobule, R precentral gyrus, R superior frontal gyrus.	L,R	28	4	48	613	4.25
	Inferior lateral occipital cortex, lingual gyrus, inferior temporal gyrus, posterior temporal fusiform cortex, posterior parahippocampal gyrus, temporal occipital fusiform cortex,	L	-34	-34	-22	542	3.75
Main effect of HYA (HYA > WMH)							
	Supracalcarine cortex, intracalcarine cortex, lingual gyrus, inferior lateral occipital cortex, occipital pole, occipital fusiform gyrus.	L	-16	-84	-2	758	3.77
	L and R precuneous cortex, L and R lingual gyrus, L and R posterior cingulate gyrus, R cuneal cortex, R supracalcarine cortex.	L, R	6	-62	4	564	3.4
**Executive contrast**							
Main effect of WMH (WMH > HYA)							
	L and R precuneous cortex, R Posterior cingulate gyrus, R cuneal cortex.	L, R	-10	-54	40	767	3.21


*Areas defined by the Harvard-Oxford Structural Atlas. L; Left, R; Right, HOA, Healthy Older Adults; WMH, White Matter Hyperintensity group; HYA, Healthy Younger Adults, MNI: Montreal Neurological Institute.*

### Association of Behavior and Weighted-Echoes fMRI Activation

**FIGURE 5:**
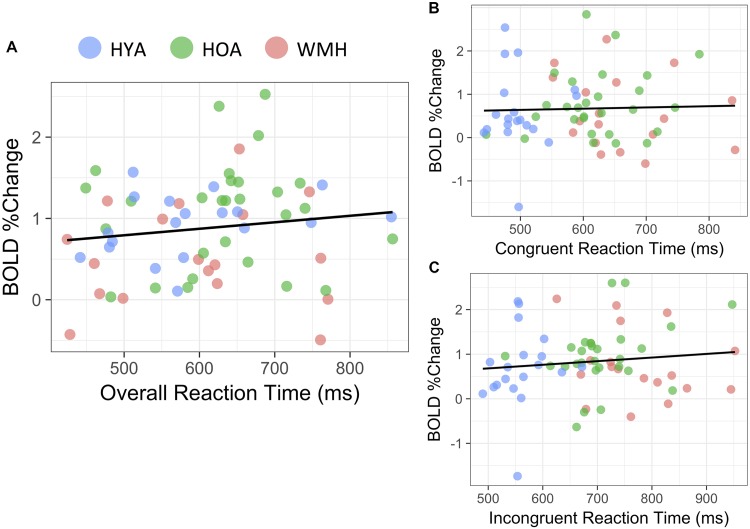
Reaction time correlation with BOLD% change. **(A)** During the *all versus baseline* condition, average reaction time was not significantly linked to BOLD% change throughout the task (*R*^2^ = 0.01, *P* = 0.2). **(B)** *Congruent* (*P* = 0.4) and **(C)** *Incongruent versus baseline* conditions similarly showed no significant correspondence to reaction time during respective conditions (*P* = 0.4).

In the HOA group, there was a mild but significant association between mean reaction time of all task conditions (i.e., incongruent and congruent) on BOLD % signal change (*R*^2^ = 0.07, *P* = 0.03 (**Figure 5A**). No associations were observed for either the WMH (*P* = 0.5) or HYA (*P* = 0.5) groups. Further, no association between BOLD % signal change and mean reaction time was observed when data was combined from all groups (*R*^2^ = 0.01, *P* = 0.2) (**Figure 5A**). Accuracy did not correlate with BOLD % signal change for any of the groups individually (*P* > 0.08) or in a combined analysis (*R*^2^ = 0.02, *P* = 0.1). Similarly, performance did not correlate to BOLD % in the combined sample when separated into *congruent* (mean reaction time: *P* = 0.8; accuracy: *P* = 0.4; **Figure 5B**) and *incongruent* conditions (mean reaction time: *P* = 0.4; accuracy: *P* = 0.4; **Figure 5C**).

### Executive Contrast Weighted-Echoes fMRI Group Activation Differences

**FIGURE 6:**
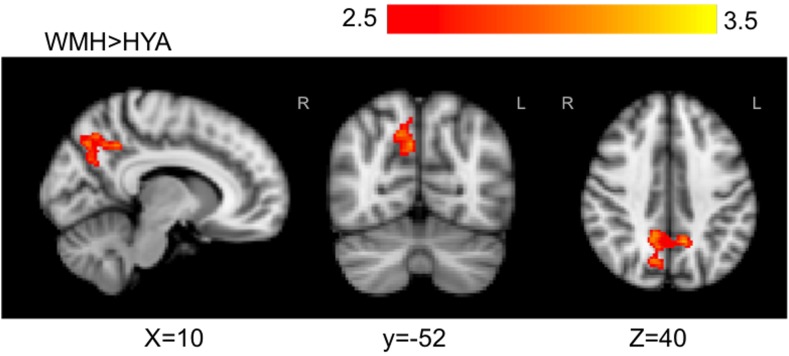
Statistically significant signal differences found in WMH versus HYA for *executive* contrast. WMH, when compared to HYA, demonstrated greater local activation bilaterally in the precuneous, and in the right posterior cingulate and cuneal cortex. Primary threshold for all maps was *Z* = 2.32 and cluster-correction threshold was *P* = 0.05. Warm colors, z-stat intensity differences.

The *executive* contrast maps, adjusted for correct-trial reaction time, for the HOA group showed mean activation in the frontal pole, superior and middle frontal gyri, paracingulate gyrus, superior parietal lobule, lateral occipital cortex, precuneous, angular gyrus, and supramarginal gyrus. For the WMH group, mean activation was observed in the angular gyrus, superior parietal lobe, precuneous cortex, postcentral gyrus, supramarginal gyrus, lateral occipital cortex, and cingulate gyrus. The HYA group did not show mean activation above threshold for *executive* contrast (data not shown). Group differences were observed in the lateral occipital lobe, superior parietal lobule, supramargial gyrus, and angular gyrus (omnibus ANOVA: *P* < 0.05). *Post hoc* pairwise group comparison revealed WMH had greater activation compared to HYA in a parieto-occipital region comprising the precuneous, posterior cingulate gyrus, and supracalcarine cortex (**Table 3** and **Figure 6**). No significant clusters were found for the reverse contrast, or any contrast involving the HOA group. Reaction-time-unadjusted *executive* contrasts showed similar average activation patterns in HOA and WMH, and a similar absence of significant voxels for this contrast in HYA. ANOVA did not reveal group differences.

## Discussion

This study implemented multi-echo BOLD fMRI during an attention-demanding test to identify brain activation differences between older adults with and without WMH, as well as young healthy adults. Adults with WMH performed worse on attention-related tasks in terms of processing speed but not accuracy. In concert with our successful efforts of achieving robust fMRI signals through weighted-echo BOLD, the WMH group had a smaller spatial extent of activation patterns compared to HOA, after adjusting for reaction time performance. Notably, individuals with WMH had lower activation in the fronto-parietal regions and temporo-parietal junction compared to HOA. In an *executive* function contrast, WMH activated areas of the precuneous and posterior cingulate gyrus more than HYA, despite similar congruency-related effects on reaction time. These results suggest WMH may contribute to disruption of functional brain networks that underlie salience and cognitively mediated attention.

We showed significant group differences using a robust multi-echo BOLD fMRI method, which is an acquisition and analysis strategy that emphasizes activation by diminishing spurious unrelated signal and increasing the contrast to noise ratio (Kundu et al., 2013, 2015, 2017; Gonzalez-Castillo et al., 2016). Recently, researchers have used similar approaches to study fMRI among healthy younger, middle-aged (Kundu et al., 2015; DuPre et al., 2016; Vértes et al., 2016; McDonald et al., 2017), and obese adults (Baek et al., 2017). Our study adds to this body of literature by applying the technique to an older cohort with inherently compromised BOLD signal-to-noise (Huettel et al., 2001). We showed that multi-echo BOLD weighting decreased the global correspondence between the denoised data and the raw data, while maintaining a wider spatial activation compared to RETROICOR (see **Supplementary Figure S1**). A lower slope in WMH using the weighted-echoes method presumably reflects greater removal of physiological and other noise sources that increase with age and/or disease (Huettel et al., 2001). Further, the effectiveness of RETROICOR was variable across groups, demonstrated by a lower correspondence, that is *R*^2^, with the single echo fMRI reference in WMH, which may introduce systematic bias to group comparisons of activation. The more nuanced multi-echo BOLD approach differs from RETROICOR’s uniform denoising technique and may be beneficial in heterogeneous cohorts where atrophy and/or aging elicit spatially varying influences on BOLD images.

The *all versus baseline* condition is of interest in the current analysis as it highlights total task-positive network activation differences between HOA and WMH that can go undetected in higher-level analyses among older adults. HYA consistently had faster processing speed than HOA without extensive BOLD activation in the current study. In particular, there was greater activation in regions of the occipito-temporal cortex in HOA compared to HYA, a region important for prediction of upcoming motor-action (Gallivan et al., 2013). This may reflect a lower task difficulty for the HYA, which others attribute to down regulation of a task-positive network (Reuter-Lorenz and Cappell, 2008). Increased recruitment of brain resources may be influenced by task difficulty in HOA; a relationship emphasized by elevated activation compared to WMH and HYA and in previous work demonstrating compensatory reallocation during cognitively demanding tasks (Cabeza et al., 2002).

One explanation for the hypoactivation of fronto-parietal and temporal regions seen in WMH, compared to HOA during *all versus baseline* condition, could be that WMH experienced relatively higher cognitive load, which led to hypoactivity within these regions coupled with slower processing speeds. More explicitly, HOA implement a compensation strategy of increased prefrontal and parietal cortex recruitment at low levels of cognitive load compared to younger adults (Mattay et al., 2006; Cappell et al., 2010; Schneider-Garces et al., 2010), however, at higher cognitive load, this strategy often fails and results in equivalent or reduced activity (Grady, 2012; Kennedy et al., 2017). Similar to our findings in WMH, greater WMH lesion volume is associated with lower activation in the left middle frontal gyrus and right posterior parietal cortex (Venkatraman et al., 2010); regions recruited during attentional tasks in healthy older and younger adults (Fan et al., 2002, 2005; Langenecker et al., 2004). The presence of WMH, therefore, may lead to differential neural network responses compared to HOA.

BOLD % signal increased as performance decreased in HOA, but this association was not found in WMH or HYA during the *all versus baseline* condition. This relationship reinforces the link between task performance and cognitive demand in HOA (Cabeza et al., 2002), and highlights a decoupling of performance and neurovascular response in WMH. Our findings are consistent with aspects of neural network theories, in which accumulation of WMH can impact gray matter resting-state/task-based functional connectivity (De Marco et al., 2017) and thus compensatory recruitment (Lockhart et al., 2015; Dey et al., 2016). Indeed, cholinergic fibers are necessary for fronto-parietal, default mode network connections (Cheng et al., 2012) and anterior-posterior coherences (Moretti et al., 2008; Babiloni et al., 2009). Damage to anterior-posterior tracts that connect dorsolateral prefrontal cortex to the intraparietal sulcus may lead to hypoactivation in WMH (this study, Venkatraman et al., 2010). Loss of inter- and intra-hemispheric connections can interfere with the lateralization and synchronization of network nodes (Marstaller et al., 2015), which in turn can explain poor cognitive performance in WMH. There are limited functional connectivity analyses explicitly in WMH; future studies using resting-state fMRI connectivity would provide important insight into this potential neural network breakdown.

The effect of congruency (i.e., *executive* condition) during the flanker task further characterized the WMH brain network profile. Our findings are consistent with evidence that executive function performance, probed by the attention network test, is equivalent in older and younger adults (Fernandez-Duque and Black, 2006; Jennings et al., 2007). We found no difference in executive contrast activation between older adult groups. The WMH group did not show activation in frontal regions that was observed in HOA (data not shown); this may reflect reduced connectivity in prefrontal regions in WMH as seen from resting-state fMRI (Schaefer et al., 2014). This pattern did not map onto executive behavioral performance, potentially due to the poor association of periventricular WMH with executive function (Prins et al., 2005). This analysis could have been limited by low statistical power, reducing the effect of group activation differences using an active baseline. The WMH group had higher BOLD response compared to HYA during the *executive* contrast, which is consistent with other studies on executive function (Gold et al., 2017) and spatial search tasks (Lockhart et al., 2015; Gold et al., 2017), and is linked with white matter tract deterioration (Madden et al., 2007; Persson et al., 2011). Unlike reports of frontal-network over-activation (Lockhart et al., 2015; Gold et al., 2017), WMH atypically recruited highly vascularized areas of the precuneous and posterior cingulate. The precuneous, posterior cingulate cortex, and surrounding regions are a core feature of the default mode network (McKiernan et al., 2003). However, the posterior cingulate-precuneous network exhibits hyper-activation during task in attention-deficit/hyperactivity disorder (Castellanos et al., 2008), and evidence points to its recruitment under specific task-positive conditions (Utevsky et al., 2014). Underlying differences in neural activation of these areas in WMH are more likely explained by impaired physiology such as vascular deterioration and subsequent hypoperfusion (Miners et al., 2016), and/or reduced glucose metabolism (Hirono et al., 2004; Choo et al., 2007; Brown et al., 2014; Miners et al., 2016).

The limitations of this study are as follows. Group differences in the *all versus baseline* fMRI signal may be influenced by alterations in the hemodynamic response, which is described in overt stroke groups but has yet to be investigated among adults with subclinical WMH (Roc et al., 2006; Blicher et al., 2012). A delayed hemodynamic response is plausible given the pathophysiological features of WMH, namely endothelial dysfunction, atherosclerosis and arteriosclerosis (Wardlaw et al., 2013). Moreover, the need to identify WMH presence *a priori*, which we addressed by pre-study screening for vascular risk factors, through a family medicine clinic mail-out, limited recruitment efforts. The current study does not separate individuals by other forms of small vessel disease such as cerebral microbleeds or enlarged perivascular spaces. Considering the Fazekas scoring system is widely used, there is clinical prudence to separating the cohorts based on this rating scheme. The current study investigated fMRI activation differences from one attention-related task, which was chosen due to attention and psychomotor speed as hallmark cognitive features of WMH; however, future studies exploiting multi-domain cognitive fMRI may provide added insight. Finally, we acknowledge that recent debates highlighting potentially high false-positive rates in fMRI studies might have bearing on single-echo BOLD data (Eklund et al., 2016; Slotnick, 2017). Methods comparison analysis in this study shows reduced signal compared to single-echo data used in such conventional fMRI studies, therefore, we in part used weighted multi-echo data to account for potentially inflated false positive rates (Lombardo et al., 2016).

## Conclusion

This study identified a dichotomy between older adults with and without WMH, characterized by brain activation differences elicited by an attention network task during multi-echo fMRI. We observed weak brain-behavior links, which subsequently fail to elucidate how neural network strategies may be applied to performance. This sets the precedent for exploring WMH-linked network dysfunction through resting-state fMRI analyses. Subsequent studies should also explore the nature of the blood-tissue transfer of oxygen in those with neurovascular pathology to further our understanding of the neurophysiological consequences of CSVD.

## Author Contributions

SA, AM, and BM conceived and designed the research. SA performed the experiments and analyzed the data. SA and BM interpreted results of experiments. SA prepared the figures and drafted the manuscript. SA and BM edited and revised the manuscript. All authors provided feedback on manuscript. SA and BM approved final version of the manuscript.

## Conflict of Interest Statement

The authors declare that the research was conducted in the absence of any commercial or financial relationships that could be construed as a potential conflict of interest.

## Abbreviations

ANOVA: analysis of variance
BOLD: blood oxygenation level dependent
CSVD: cerebral small vessel disease
DSCT: Digit Symbol Coding Test
FEAT: FMRIB Expert Analysis Tool
FLAIR: Fluid Attenuated Inversion Recovery
fMRI: functional magnetic resonance imaging
HOA: healthy older adults
HYA: healthy younger adults
MNI: Montreal Neurological Institute
MoCA: Montreal Cognitive Assessment
MRI: magnetic resonance imaging
RETROICOR: Retrospective Correction Technique
S: Signal Intensity
T2^∗^: relaxation time
TE: echo time
TR: repetition time
WMH: white matter hyperintensities
