# Supplementary material for: Attention-Related Brain Activation Is Altered in Older Adults With White Matter Hyperintensities Using Multi-Echo fMRI
Source: Front Neurosci. 2018 Oct 18;12:748. doi: 10.3389/fnins.2018.00748 (PMC6200839; doi:10.3389/fnins.2018.00748)
Supplement: Supplementary file 1 [file Image_1.PDF]

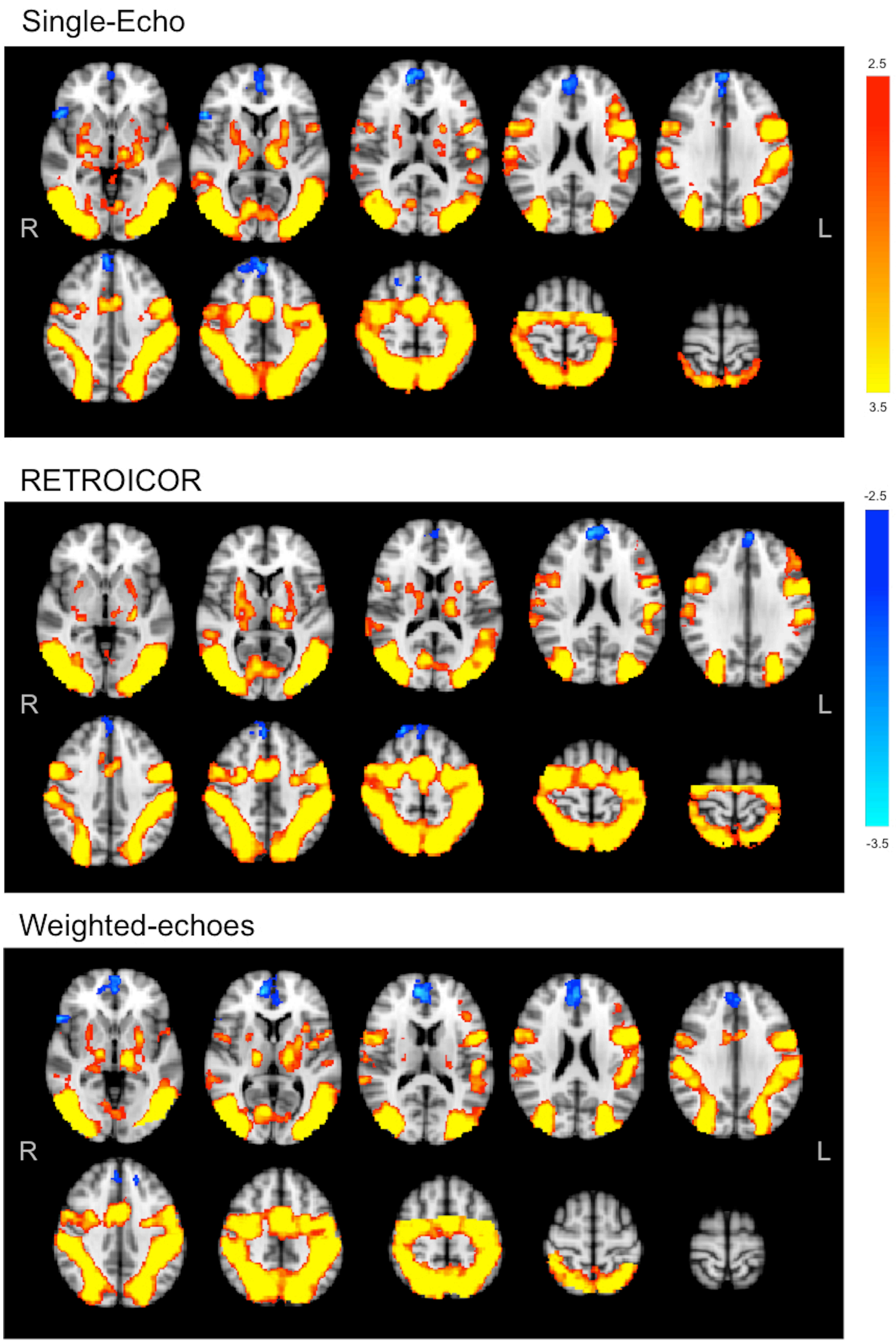

Supplementary Figure S1: Group average statistical maps of BOLD fMRI signal using single-echo, RETROICOR, and weighted-echoes image processing. The utility of multi-echo fMRI was examined by comparing the weighted-echoes and single-echo RETROICOR approaches to the uncorrected single-echo data. Using single-echo functional images during *all versus baseline* contrast, brain regions with negative z-statistics (cool colors) and positive z-statistics (warm colors) was found in the occipital lobe, parietal, and frontal fields. The use of weighted-echoes appears to show regionally relevant activation, similar to RETROICOR method.
